# Bio-Insecticidal Nanoemulsions of Essential Oil and Lipid-Soluble Fractions of *Pogostemon cablin*

**DOI:** 10.3389/fpls.2022.874221

**Published:** 2022-04-29

**Authors:** Keerthiraj Manjesh, Aditi Kundu, Anirban Dutta, Supradip Saha, Bhagyasree Sira Neelakanthaiah

**Affiliations:** ^1^Division of Agricultural Chemicals, ICAR-Indian Agricultural Research Institute, New Delhi, India; ^2^Division of Entomology, ICAR-Indian Agricultural Research Institute, New Delhi, India

**Keywords:** biopolymer, volatile oil, ultrasonication, acaricidal, patchouli, *Tetranychus urticae*

## Abstract

The present study aimed to develop nanoemulsions (NEs) of essential oil (EO) and lipid-soluble extract (HE) of *Pogostemon cablin* leaves using biosurfactant, saponin. Hydro-distilled EO and fat-soluble HE were analyzed using GC-MS, which revealed 38.7 ± 2.7% and 37.5 ± 2.1% patchoulol, respectively. EO and HE were formulated with saponin to prepare corresponding coarse emulsions (CEs); furthermore, high-speed homogenization for 2 min was followed by ultrasonication for 3 min with constant frequency of 50 kHz. of the CEs resulted in respective NEs. NEs were characterized for the physico-chemical properties such as emulsion intrinsic stability, particle size distribution, polydispersity index (PDI), and transmission electron microscopy (TEM) for morphology and accurate nanodroplet diameters. CEs and NEs were investigated for insecticidal efficacy against adults of *Tetranychus urticae* and larvae of *Spodoptera litura*. Stable NEs of EO and HE at 500 μg mL^−1^ concentration exhibited corresponding average particle size of 51.7 and 89.9 nm, while TEM image revealed spherical-shaped droplets with the average droplet diameters of 15.3 and 29.4 nm, respectively. NEs of EO and HE displayed highest efficacy in contact toxicity (LC_50_ 43.2 and 58.4 μg mL^−1^) after 48 h and fumigant toxicity (LC_50_ 9.3 and 13.6 μg mL^−1^) after 24 h against *T. urticae*. In addition, NEs of EO showed considerable antifeedant and feeding deterrent action (AI 99.21 ± 0.74 and FI 99.73 ± 1.24) against *S. litura* larvae.

## Introduction

The present concept of green crop protection tools emphasized the exploitation of bioactive volatile and non-volatile phyto-constituents, which serve as potential sources of new molecules with a complex mechanism of action (Pavela and Benelli, 2016). In spite of huge versatility of the natural compounds, common constraints exist for their delivery systems, which are related to limited aqueous solubility and stability. Nanotechnological interventions are represented as one of the promising solutions of the problem (Nenaah et al., 2015; Campolo et al., 2017). Further increasing interests have been focused in recent studies regarding the development of NEs for encapsulation of volatile bioactive compounds/EOs for their promising application in agriculture (Khot et al., 2012). EOs have been recognized as eco-benign promising crop protection tool (Kundu et al., 2021); thus, nano-formulations of EOs are being designed, developed, and evaluated to manage many economically important pests (Heydari et al., 2020).

NEs provide a structural framework where bioactive ingredients are dispersed in the aqueous medium and stabilized with the help of surfactant particles. The stable oil-in-water NEs usually comprised of lipophilic active ingredient(s), surfactant, and water (Noori et al., 2018). It appears mostly transparent or slightly translucent in texture with the particle diameter within 100 nm (Balasubramani et al., 2017), since Sugumar et al. (2014) considered NEs having droplet size ranging between 20 and 200 nm. High-pressure homogenization has been used to prepare emulsions with reduced diameter; however, the process could only be sustained on high consumption of energy. Micro-fluidization is another energy-intensive technique, which shears the emulsion droplet size through molecular collision under microfluidic compartment (McClements, 2004). Related studies on the preparation of NEs using various techniques have indicated ultrasonic energy as competitive or even relatively superior employing rotor–stator dispersing to achieve uniform nanodroplets (Kentish et al., 2008).

Nano-sizing of lipophilic components including EOs helps to form kinetically stable emulsion with improved dispersibility in aqueous medium and higher degree of aqueous diffusion (Hashem et al., 2018). Furthermore, bioavailability improved as NEs with the increased surface area dimensions could easily penetrate the cell wall and reach the specific target binding sites; therefore, nano-emulsification reduced the application rate of active ingredients (Acevedo-Fani et al., 2015). However, comprehensive investigations are truly imperative to ascertain proper encapsulation and stability of nanodroplets. Thus, emulsifiers play a crucial role, and the critical micelle concentrations (CMC) of emulsifiers usually determine the kinetic stability of the emulsion (Kumar et al., 2019a). Naturally occurring green emulsifiers such as biosurfactants, amphiphilic proteins, and polysaccharides have been exploited to prepare nanoemulsions. Biosurfactants like saponins are preferred as these are required in small quantities to develop stable nanodroplets of lipophilic compounds (McClements and Gumus, 2016). Besides, steroidal saponins have been reported to possess insecticidal properties (Dolma et al., 2021).

*Pogostemon* species are perennial herbaceous plants, which belong to Lamiaceae family, are native to Philippines, and are widely distributed across warm and humid tropical climate of South Asian countries including India (Kusuma et al., 2018). Volatile EO of *P. cablin* is primarily constituted with sesquiterpenes, namely, patchoulene and patchouli alcohol (Sundaresan et al., 2012). Investigations on biological properties of the *Pogostemon* EO and phytochemicals revealed multidimensional pharmacological functions against a panel of targets (Hu et al., 2018; Roshan et al., 2022). Significant antifeedant activity of the EO has also been recorded against cosmopolitan pests (Huang et al., 2014). Furthermore, the oil was reported to exhibit LD_50_ 0.2 μg/adult against *Tribolium castaneum* (Feng et al., 2019) and LD_50_ 8.0 μg/insect against *Choristoneura rosaceana* (Machial et al., 2010). Based on the assumptions of higher efficacy of EO and extracts of *Pogostemon*, patchouli alcohol appeared as the key component responsible for broad-spectrum activities (Lima et al., 2013).

Hence, the hypothesis has been built with the proof of concept to utilize the EO and lipid-soluble fractions of *P. cablin* for the preparation of NEs with improved efficacy against acarid and insect. With these backgrounds, the present research was designed to profile volatile chemical constituents of EO and lipid-soluble fractions of *P. cablin* for the development of NE-based delivery system in an attempt to achieve potential bio-insecticide.

## Materials and Methods

### Plant Materials

Fresh leaves of *P. cablin* (5.0 kg) were collected from farmer's field, Hirisave village (12.9172° N and 76.4563° E) near Hassan district of Karnataka, India, during the month of April 2019. The voucher specimen (PC-2019-KHV-01) was authenticated from ICAR-National Bureau of Plant Genetic Resources, New Delhi, India. Fresh leaves were cleaned, gently washed with water, and used for isolation of EO. Shade dried leaves were powdered and used for extraction.

### Distillation of EO

Fresh leaves of *P. cablin* (1.0 kg) were hydro-distilled in a Clevenger's apparatus (Borosil Glass Works Ltd., Mumbai, India) for continuous 12 h according to the method reported by Kundu et al. (2016). Pale yellowish-colored EO was collected from the apparatus. Furthermore, EO was partitioned with diethyl ether (3 × 50 mL) followed by passing through anhydrous sodium sulfate (20 g) using a glass funnel and stored. The yield of EO (%) was calculated as 1.43% (v/w).

### Extraction

Coarsely powdered leaves (1.0 kg) of *P. cablin* were submerged with 2.5-L hexane (Merck® India Ltd, Mumbai, India) and sonicated for 2 h at 35°C using bath sonicator (PCI Analytics Ltd, Mumbai, India) following the method reported by Dutta et al. (2021). The extraction was repeated thrice with the same sample followed by filtration and concentrated to dryness under reduced pressure in a rotary evaporator (Heidolph, Germany) below 40°C to afford the crude HE (109.7 g).

### GC-MS Analysis

*Pogostemon* EO and HE were analyzed in a 5590C GC-MS (Agilent Technologies®, USA) using a stationary phase column (30 m × 0.25 μm, 0.25 μm, Agilent Technologies®, USA) which was equipped to a mass spectrometer. Samples (1 μL, each) were injected through auto-injector under split-less mode. Helium was used as carrier gas with the flow rate of 1 mL min^−1^ and pressure of 10 psi. Then, oven condition was programed where temperature started at 30°C held for 1 min., then increased at the rate of 3°C min^−1^ to reach 60°C, and then held for 5 min. Hereafter, temperature was increased with the rate of 2°C min^−1^ to reach 150°C and with the hold time of 5 min. Next, temperature was again raised at the rate of 5°C min^−1^ to reach 220°C with the hold time of 5 min. At last, temperature increased to 280°C at the rate of 10°C min^−1^. Both the samples were analyzed with the runtime of 90 min. The MS acquisition parameters were programed with the ion source temperature of 170°C, electron ionization of 70 eV, transfer line temperature of 280°C, solvent delay of 3 min., and E.M. voltage of 1,419 V. The ionization energy (70 eV) was fixed with scanning rate of 1 s with the mass range of 50–550 amu. Volatile aromatic constituents were identified by matching their mass spectra, fragmentation pattern, reference standard, and retention index (literature and experimental) using Adams (2007), NIST, and WILEY libraries (Kumar et al., 2021).

### Critical Micelle Concentration

Saponin (C_36_H_54_O_11_, sapogenin content 20–35%) sourced from *Quillaja* sp. (Merck® India Ltd. (Mumbai, India) was used as biosurfactant. CMC of saponin was determined from electrical conductance (EC) of different concentrations. For that, 100 to 12,000 μg mL^−1^ of saponin was prepared in aqueous medium and EC values were determined by a probe-type waterproof EC meter (HI 98304, Hanna, New Delhi, India). At first, the EC meter probe was calibrated with the ready-made KCl solution of known strength. Then, the prepared saponin aqueous solutions were measured by stirring and maintaining temperature equilibrium at 25 ± 1°C. The probe was washed thoroughly with de-ionized water after each measurement, starting from lower to higher concentrations of saponin.

### Nanoemulsions (NEs) Preparation

At first, primary coarse emulsions (CEs) of EO and HE of *P. cablin* were prepared separately with the double concentration of pre-determined CMC values of saponin (0.5%). To prepare the CEs, EO (0.5g) and HE (0.5g) were separately mixed with the surfactant, and saponin (0.25 g) with minimum amount of deionized water, and finally, the volume was made up to 50 mL and vortexed for 5 min to obtain CEs (1%) of EO and HE. The freshly prepared CEs were thoroughly dispersed individually in high-speed homogenizer (IKA Ultra-Turrax T25, India) for 2 min. Thereafter, emulsion dilution technique was used to prepare nanoemulsions (NEs) from the CEs (Ghosh et al., 2013). Both the CEs (1%) were diluted serially with 0.5% aqueous solution of saponin to prepare secondary emulsions of lower concentrations (31.25–500 μg mL^−1^) of EO and HE. The diluted secondary emulsions were then subjected to ultrasonication using a probe ultrasonicator (MISONIX, Ultrasonic Liquid Processors, USA) for 3 min. at the amplitude of 50 kHz. to obtain NEs.

### Stability of NEs

NEs of EO and HE were subjected to centrifugation at 10,000 rpm for 20 min. for checking any phase separation. Furthermore, the stability of the prepared emulsions was observed at room temperature at different time intervals and at accelerated storage condition (54 ± 1°C) for 14 days.

### Particle Size of NEs

Average particle diameter, distribution, zeta potential, and polydispersity index (PDI) of the NEs and CEs of EO and HE were determined using a Zetasizer (Microtrac, Germany) following the principles of dynamic light scattering. Samples were measured with the help of a probe attached with the instrument with a laser light source. Microtrac FLEX data analysis program was used for the measurement of average droplet size. All the measurements were replicated thrice for each concentration.

### Transmission Electron Microscopy

NEs were visualized using TEM (JEM 1011, JEOL, Japan) operated at an acceleration voltage of 80 kV to determine the morphology and droplet size at all the concentrations. The Cu-coated grid (200 mesh) of TEM was impregnated with each concentration of NE and kept for 15 min. for partial drying. The grids were further stained with 2% uranyl acetate and allowed to dry again for 3 h, and micrographs were acquired at the magnification of 80,000x at 100 nm under TEM.

### Acaricidal Assays

#### Culturing of *T. urticae*

Adults of *Tetranychus urticae* were collected from tomato ecosystem which had not been exposed to any acaricide before. The acarids were reared on surface of the mulberry (*Morus alba*) leaves and kept on wet sponge in the laboratory at 27 ± 1°C, 65 ± 5% RH, and 13:11 h under L:D photoperiod till three generations before conducting the bioassay to obtain pure culture. Mites took 8–10 days during summer and 10–16 days during winter to complete one generation.

#### Leaf Dip Assay

CEs and NEs were tested against adults of *T. urticae* following leaf dip method. Test samples at the strength of 31.25–500 μg mL^−1^ were used for acaricidal assay. Mulberry (*Morus alba*) leaves were cleaned and treated separately with different concentration of the samples. Treated leaves were allowed to dry for 2 h, and 25 adults of *T. urticae* were transferred to the treated leaves. Saponin solution was used as negative control. All the Petri plates were incubated at ambient laboratory conditions under insect culture chamber maintaining 27 ± 1°C. Observations were taken after 24- and 48-h exposure. Mortality (%) was calculated, and probit analysis and LC_50_ values (μg mL^−1^) were determined using statistical software.

#### Fumigation Assay

CEs and NEs were tested for fumigant toxicity against adults of *T. urticae*. Using a stereomicroscope, 25 adults were transferred on the cleaned mulberry leaf holding on the dorsal part of the hysterosoma using a handling brush. The leaves were kept inside the glass jars. Each treatment (2.0 mL) of each sample was socked in cotton balls and hung with the help of lid inside the jar. Test concentrations of each sample were kept at 100–5.0 μg mL^−1^. Each treatment was replicated five times along with negative control, and the dead adults were counted after 24 h. The treated adults were considered dead if appendages did not respond even after touching with the brush. Mortality (%) was recorded, and further, LC_50_ values (μg mL^−1^) were determined.

### Insecticidal Activity Against *S. litura*

#### Insect Culture

Eggs of *S. litura* were collected from tomato plants and incubated under laboratory conditions with high RH of 80 ± 5%. When eggs turned into dark color, matured (3–4 days), and were inoculated for hatching, freshly hatched larvae were fed on castor (*Ricinus communis*) leaf bouquets till larvae entered into pupation. Then, sex was identified, separated in different jars for emergence, and released for oviposition in jar, which consists of 15–20% honey solution dipped in a cotton wad for food and zig-zag folded paper strip for egg laying. Field collected cultures were reared in laboratory for 2–3 generations to obtain pure culture and used for testing.

#### Larvicidal Assay

Larvicidal activity of the CEs and NEs based on EO and HE was studied using potter tower spray at five different test concentrations (500–31.25 μg mL^−1^). Fresh castor leaf disk was kept in each Petri dish, and five third instar larvae of *S. litura* were released. Each test concentration (1.0 mL) was sprayed on the third instar larvae of *S. litura* kept in Petri dish. Each treatment was replicated thrice. Petri dishes were kept under laboratory ambient conditions, and observations were taken after 24 and 48 h. Mortality (%) was calculated, and lethal concentration in terms of LC50 values (μg mL^−1^) was determined.

#### Antifeedant Assay

CEs and NEs were evaluated using non-choice and choice leaf disk method (Sengonca et al., 2006). Briefly, fresh castor leaves were collected from field and cleaned thoroughly. Leaves were cut evenly maintaining disk desired size (3 × 3 cm^2^), were dipped in various test concentrations (500–31.25 μg mL^−1^) separately, and were allowed to air dry at room temperature for 3 h. Additionally, leaf disks dipped in saponin solution were used as negative control. In each Petri dish, one layer of wet filter paper was placed to avoid drying of the leaf disks, if any. One third instar larva was introduced into each treated plate and placed in an incubator at 27 ± 1°C with 65 ± 5% RH and a 14:10 (L: D) photoperiod. Each treatment was replicated eight times. Observation of the larvae was taken after 24-h exposure to determine the effect of on their feeding behavior. Larvae were found to be sterile and could not feed the leaves; however, most of the treated plates, close to complete consumption of leaves, were observed in control. Feeding of treated and control leaves was measured after 24 h. using a Leaf Area Meter (ADC Bioscientific Ltd., India), and the antifeedant index (AI) was determined by the following equation AI% = [(1 – T/C) × 100], where T is the average area of treated leaf consumed and C is the average leaf area consumed without treatment. The Feeding Index (FI) was calculated as [(C – T)/(C + T)] × 100.

### Statistical Analysis

Data were measured using the Statistical Package for the Social Sciences (SPSS, Version 14.0, IBM, NY, USA). The results were expressed as mean±standard deviation (SD), and differences between variables were tested using one-way ANOVA. Statistically significant level was determined at *p*-value < 0.05.

## Results

### Volatile Composition of EO and HE

Volatile constituents of EO of *P. cablin* leaves were identified in GC-MS which showed several peaks, corresponding to twenty-four mono- and sesquiterpenoids, representing 96.9% of the oil (Table 1). Sesquiterpene hydrocarbons (55.5%) were most abundant followed by oxygenated sesquiterpenes (40.6%). Patchouli alcohol (38.7 ± 2.7%) was found as the major oxygenated sesquiterpene followed by α-bulnesene (18.1 ± 1.2%) and α-guaiene (17.7 ± 1.2%). Other major sesquiterpenes of the oil were *trans-*β-caryophyllene (3.7 ± 0.5%), β-patchoulene (3.6 ± 0.5%), β*-*elemene (3.3 ± 0.4%), β-guaiene (3.2%), α-patchoulene (2.6 ± 0.3%), and α-salinene (2.5 ± 0.2%). Only three monoterpene hydrocarbons such as α-pinene (0.2 ± 0.0%), β-pinene (0.3 ± 0.1%), and *dl*-limonene (0.2 ± 0.0%) were identified accounting only 0.7% of the EO.

**Table 1:** Chemical composition of volatile organic components of EO and HE of *P. cablin* leaves.

**^a^Compounds**	**^b^RI^exp^**	**^c^RI^lit^**	**^d^RA (%)**		**^e^Identification**

			**EO**	**HE**	
*α-*Pinene	928	932	0.2 ± 0.0	–	RI, MS
β-Pinene	969	974	0.3 ± 0.1	–	RI, MS
*dl*-Limonene	1,018	1,024	0.2 ± 0.0	–	RI, MS
Nonanal	1,103	1,105	–	3.0 ± 0.4	RI, MS
Tridecane	1,302	1,308	–	0.6 ± 0.1	RI, MS
*trans-β*-Caryophyllene	1,409	1,412	3.7 ± 0.5	4.3 ± 0.5	RI, MS
γ-Elemene	1,430	1,436	3.3 ± 0.4	0.5 ± 0.1	RI, MS
*α-*Guaiene	1,436	1,440	17.7 ± 1.2	12.4 ± 0.9	RI, MS
Aromadendrene	1,438	1,439	0.5 ± 0.1	-	RI, MS
*β-*Patchoulene	1,441	1,443	3.6 ± 0.5	0.2 ± 0.0	RI, MS
*α-*Patchoulene	1,452	1,457	2.6 ± 0.3	3.6 ± 0.5	RI, MS
Seychellene	1,458	1,460	-	2.7 ± 0.3	RI, MS
α-Selinene	1,474	1,475	2.5 ± 0.2	0.1 ± 0.0	RI, MS
γ-Gurjunene	1,478	1,479	0.1 ± 0.0	-	RI, MS
β-Selinene	1,481	1,487	0.2 ± 0.0	-	RI, MS
β-Guaiene	1,482	1,490	3.2 ± 0.4	2.5 ± 0.3	RI, MS
*α-*Bulnesene	1,509	1,505	18.1 ± 1.2	7.8 ± 0.7	RI, MS
Globulol	171	1,575	0.1 ± 0.0	–	RI, MS
Caryophyllene oxide	1,575	1,578	0.4 ± 0.1	–	RI, MS
Viridiflorol	1,607	1,612	0.5 ± 0.2	–	RI, MS
Cubenol	1,634	1,642	0.2 ± 0.0	–	RI, MS
Patchouli alcohol	1,677	1,680	38.7 ± 2.7	37.5 ± 2.1	std, RI, MS
*(Z,Z)-*Farnesol	1,721	1,718	0.2 ± 0.0	–	RI, MS
Leden oxide (I)	1,876	1,890	0.6 ± 0.1	–	RI, MS
Hexadecanoic acid	1,958	1,964	–	2.1 ± 0.2	RI, MS
Octadecenoic acid	2,147	2,140	–	6.4 ± 0.5	RI, MS
Octadecanoic acid	2,186	2,188	–	2.4 ± 0.3	RI, MS
Docosane	2,213	2,208	–	2.3 ± 0.3	RI, MS
Tetracosane	2,395	2,402	–	2.1 ± 0.3	RI, MS
Squalene	2,837	2,829	–	1.3 ± 0.2	RI, MS
Non-acosane	2,891	2,900	–	0.8 ± 0.2	RI, MS
Tricontane	2,989	3,000	–	0.9 ± 0.2	RI, MS
Total identified (%)			96.9	93.5	
*Classified on functional groups*			
Monoterpene hydrocarbons (%)	0.7	–	
Sesquiterpene hydrocarbons (%)	55.5	34.1	
Oxygenated sesquiterpenes (%)	40.6	38.7	
Aldehydes	–	3.0	
Long chain fatty acids	–	10.9	
Long chain hydrocarbons	–	5.0	

a *Compounds are listed in order of their elution from a HP-5MS column*.

b *Retention index on HP-5MS column, experimentally determined using homologous series of C_8_-C_30_ alkanes*.

c *Retention index taken from Adams (2007), NIST (2012) and WILEY libraries*.

d *Relative area % values are expressed as means ± SD*.

e *Identification methods: std, based on comparison with reference standard; RI, based on comparison of calculated RI with those reported in Adams and NIST; MS, based on comparison with WILEY and NIST 12 MS databases*.

**Figure 1:**
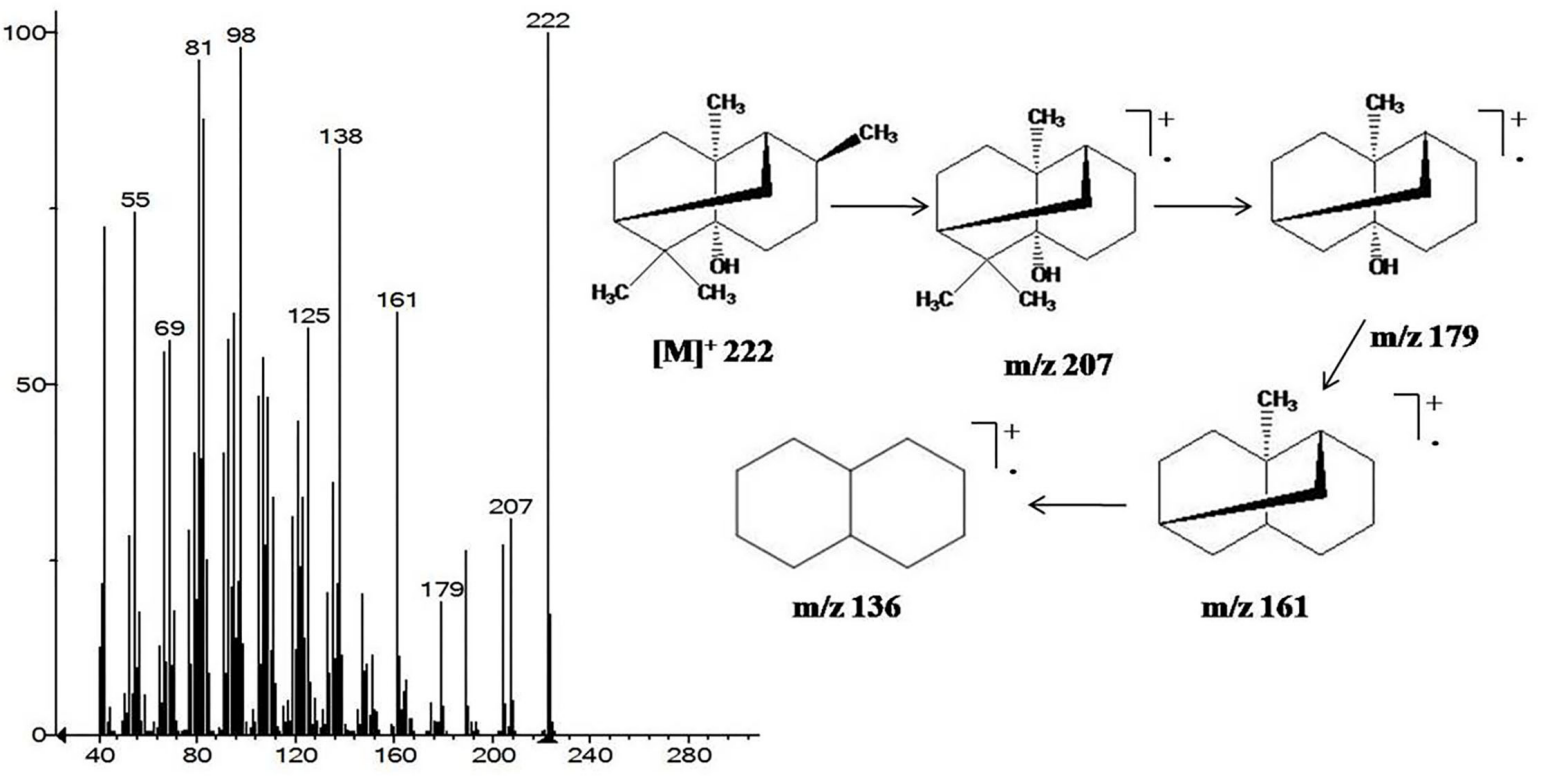
GC-MS fragmentation pattern of the most abundant patchouli alcohol characterized in EO and HE of *P. cablin*.

Mass spectrum of patchouli alcohol showed molecular ion [M]^+^ peak at m/z 222, which was further broken to give daughter ion peaks at m/z 207, 179, and 161 after sequentially losing methyl and hydroxyl moieties. Other peaks at m/z 138, 125, 98, 81, and 69 were also originated due to subsequent cleavage of hydrocarbons (Figure 1). Similarly, α-bulnesene was characterized from its characteristic [M]^+^ peak at m/z 204 and further fragmented to daughter ion peaks at m/z 189, 175, 161, 147, 135, 121, 107, 93, and 79 with removal of methylene and methyl groups (Figure 1).

Nineteen aromatic compositions, representing 93.5% of the non-polar fat-soluble HE, have been identified in GC-MS and mentioned as per their elution in HP-5MS stationary phase (Table 1). However, total sesquiterpene hydrocarbon content of hexane soluble fraction was 34.1%, while oxygenated sesquiterpene was 38.7%. Most abundant patchouli alcohol (37.5 ± 2.1%) was identified as the sole oxygenated sesquiterpene. Besides, long-chain fatty acids and long-chain hydrocarbons were found in the HE, representing 10.9 and 5.0%, respectively. Among fatty acids, octadecenoic acid (6.4 ± 0.5%) was identified as the major compound followed by octadecanoic acid (2.4 ± 0.3%) and hexadecanoic acid (2.1 ± 0.2%). Similarly, long-chain hydrocarbons, mainly docosane (2.3 ± 0.3%), tetracosane (2.1 ± 0.3%), squalene (1.3 ± 0.2%), triacontane (0.9 ± 0.2%), and non-acosane (0.8 ± 0.2%), were identified.

### Characterizations and Stability of NEs

**Figure 2:**
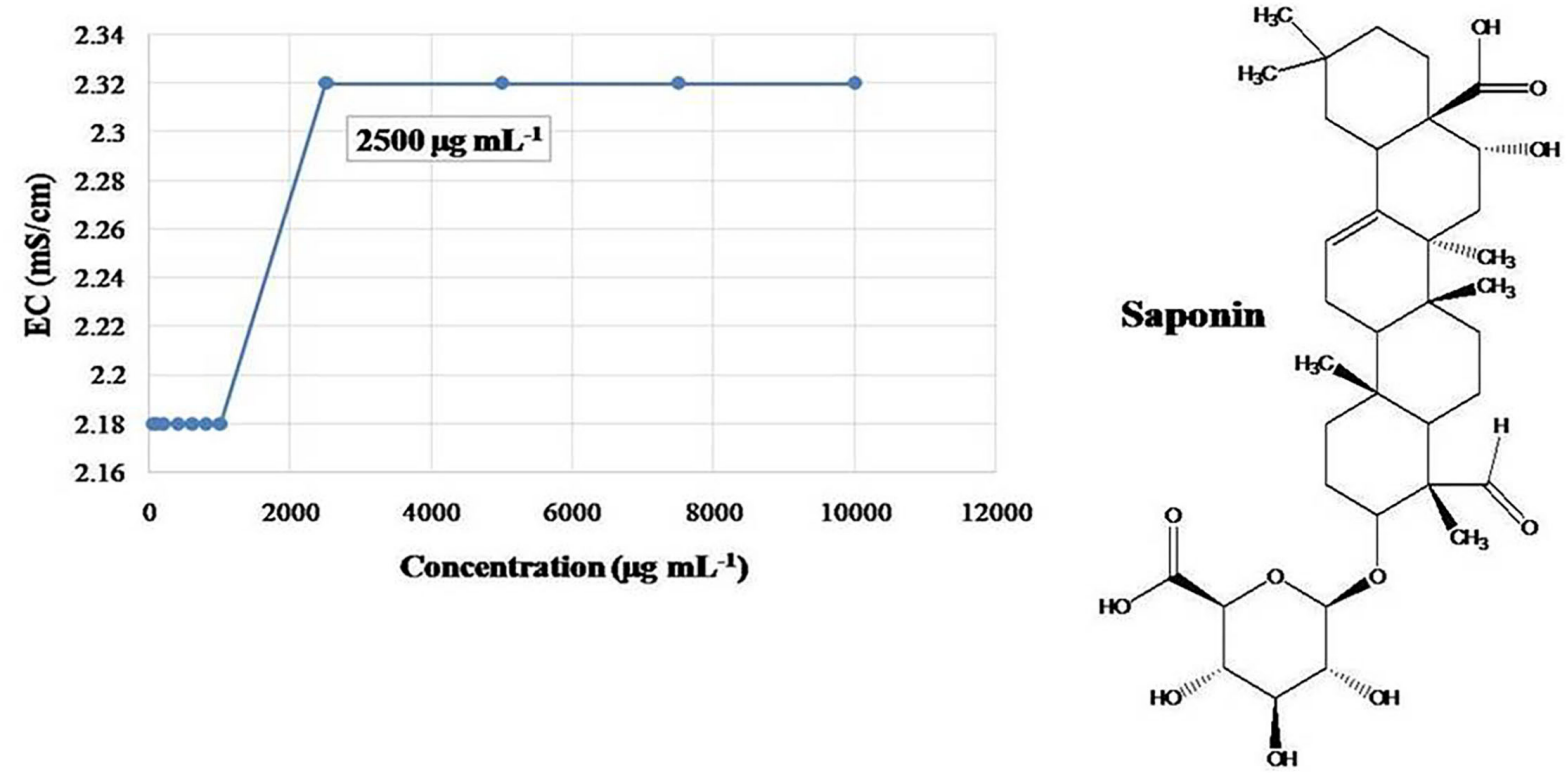
Critical micelle concentration (CMC) of biosurfactant, saponin as measured by electrical conductance (EC).

**Figure 3:**
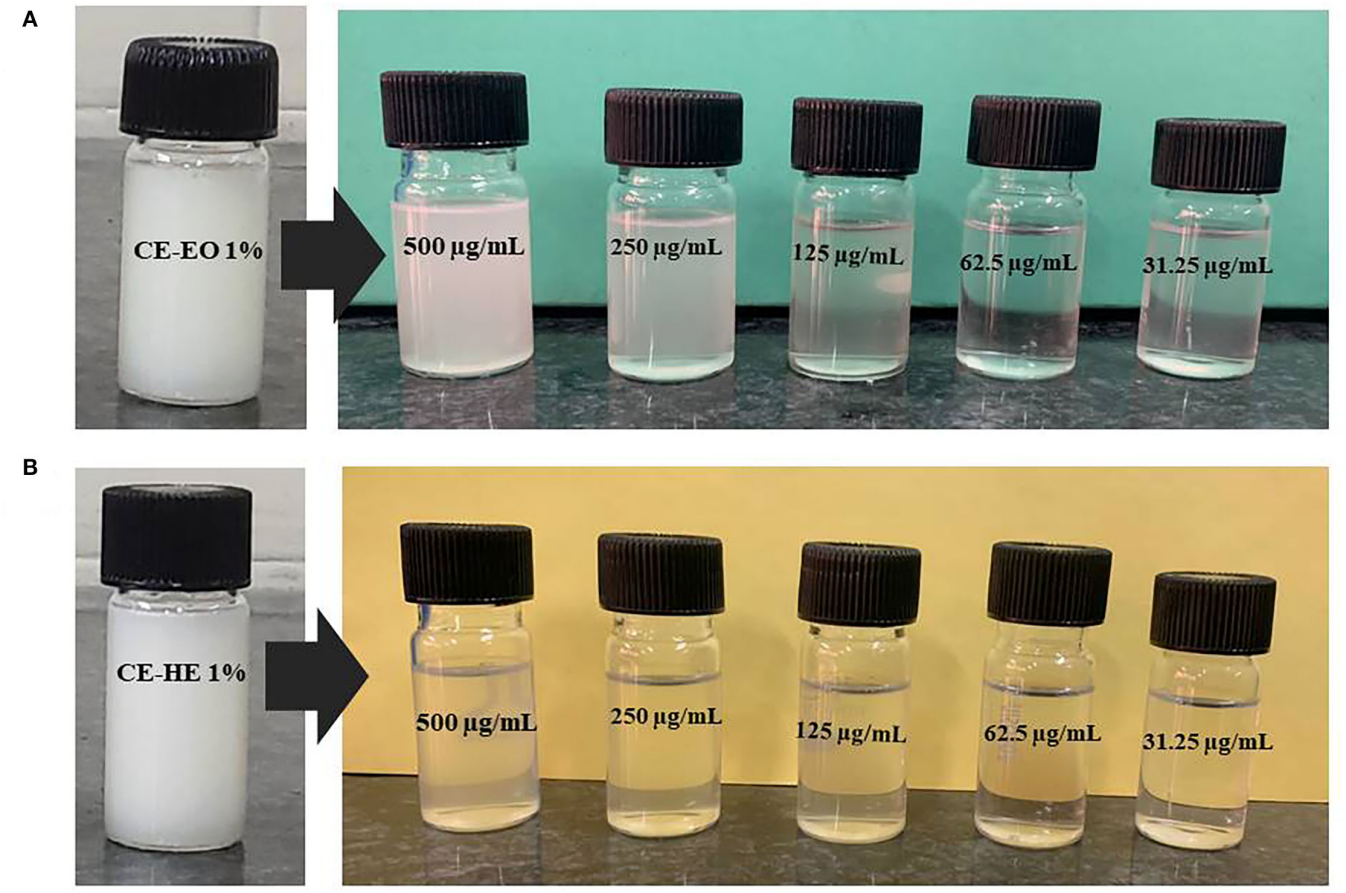
Visual appearance of CEs and different concentrations of NEs of **(A)** EO and **(B)** HE of *P. cablin*.

Based on the recorded electrical conductance of various concentrations of saponin, sharp change was observed at 0.25% (Figure 2); thus, double concentration, 0.5%, has been selected for the final preparation of CEs and NEs. Primary CEs with the 1% strength of EO (w/w) and HE were prepared separately and diluted to get various test concentrations (500–31.25 μg mL^−1^). Subsequently, ultrasonication-assisted nano-emulsification of CEs of EO and HE in aqueous medium resulted in the preparation of respective NEs (Figure 3). Formation of cavitations in the liquid due to ultrasonic wave helped to utilize the energy for shearing of coarse droplets in nano size range which was further stabilized by the surfactant particles. The prepared NEs (31.25 to 125 μg mL^−1^) were found transparent; however, slight turbidity was recorded at 250–500 μg mL^−1^ concentrations. Here, non-toxic biosurfactant, saponin, was effectively used to stabilize the developed NEs at 0.5% concentration.

**Figure 4:**
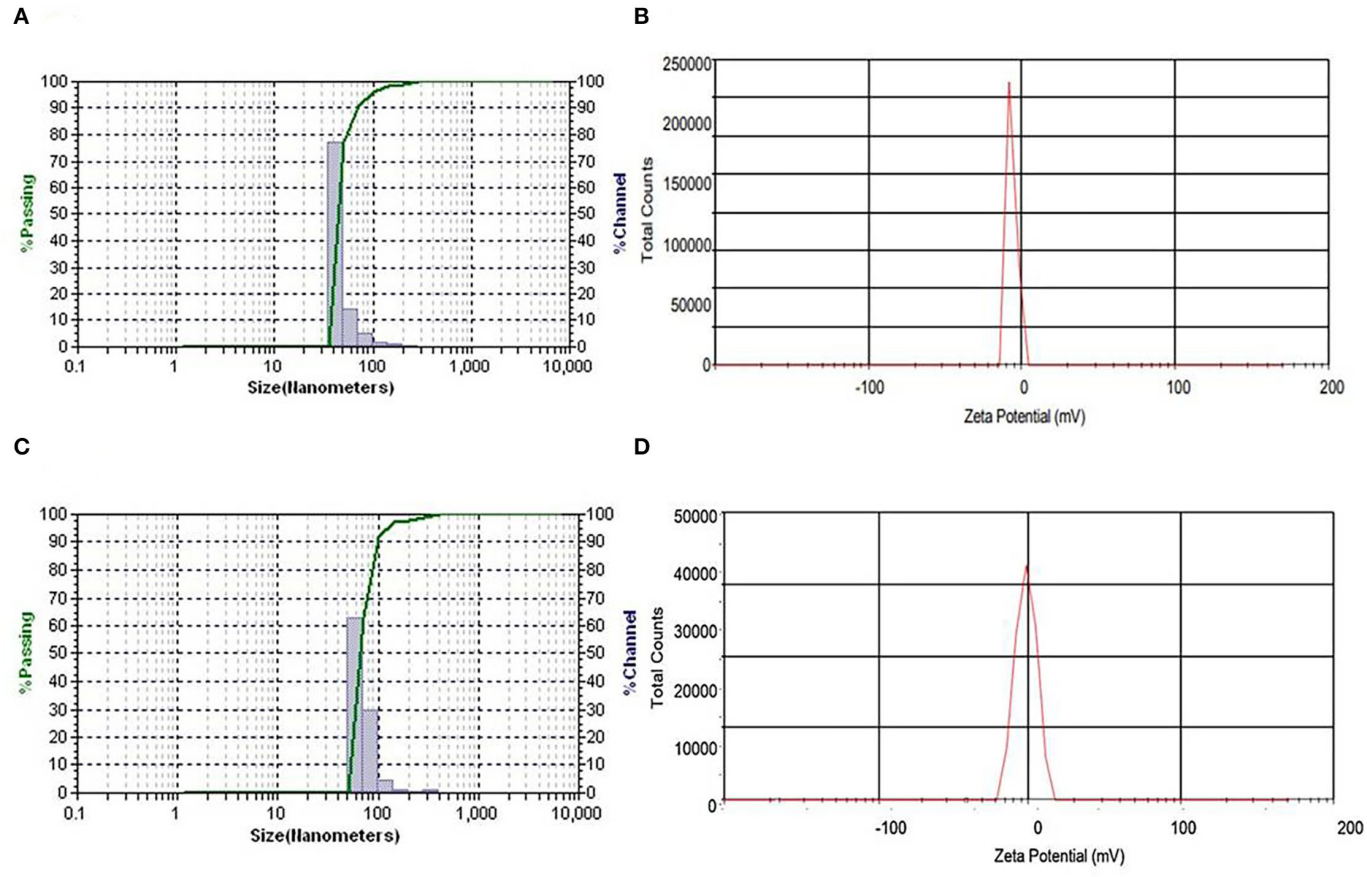
**(A)** Particle size distribution and **(B)** zeta potential of 500 μg mL^−1^ concentration of NEs of EO. **(C)** Particle size distribution and **(D)** zeta potential of 500 μg mL^−1^ concentration of NEs of HE.

The properties of serially diluted NEs of EO and HE are shown in Table 2. The average droplet diameter at 500 μg mL^−1^ concentration of NE of EO as prepared by homogenization followed by ultrasonication was 71.68 ± 0.84 nm with the polydispersity index (PDI) of 0.49 ± 0.02, signifying narrow dimension of particle size distribution (Figure 4A). Likewise, average droplet diameter at the same concentration of NE-HE was 89.87 nm (Figure 4C) with corresponding PDI 0.51 ± 0.03. There was a clear indication that with decrease in concentration of EO and HE in the NEs, mean droplet size decreased. However, no significant relationship was observed between concentration and PDI as a narrow spectrum was maintained for PDI both in case of NEs of EO (0.49–0.69) and HE (0.51–0.67), suggesting uniform size distribution of the droplets irrespective of change in concentration. As 0.5% saponin concentration was maintained throughout the study for all the samples, there was always less chance of much variation in size distribution as the micelles remained same. However, with increased loading of EO and HE, the micelles got swelled by entrapment of EO and HE. Therefore, the mean droplet diameter was found to be more at higher loading concentrations. In the present study, zeta potential of the prepared NEs of EO and HE at 500 μg mL^−1^ was found to be −29.21 ± 0.49 mV and −29.06 ±0.21 mV (Figures 4B,D) at the native P^H^ of 4.58 and 4.18, respectively. Thus, the absolute droplet charges were found very less. For all the samples, zeta potential was found to be higher than −20 mV, suggesting formation of stable NEs.

**Table 2:** Physico-chemical characterizations of NEs loaded with EO and HE of *P. cablin* leaves.

**Concentrations (μg mL^−1^)**	Mean droplet diameter (Z-average) (nm)^*^	**Zeta potential (mV)**	**Polydispersity index (PDI)**	**P^H^**
*NE-EO*				
500	71.68 ± 0.84	−29.21 ± 0.49	0.49 ± 0.02	4.58
250	62.25 ± 0.30	−27.42 ± 0.12	0.62 ± 0.02	4.95
125	58.43 ± 076	−23.49 ± 1.52	0.55 ± 0.03	5.28
62.5	57.82 ± 0.67	−23.03 ± 0.91	0.52 ± 0.03	5.99
31.25	46.19 ± 0.75	−22.62 ± 0.12	0.69 ± 0.06	6.40
*NE-HE*				
500	89.87 ± 0.62	−29.06 ±0.21	0.51 ± 0.03	4.18
250	67.93 ± 0.64	−29.18 ± 2.34	0.57 ± 0.05	4.59
125	61.40 ± 0.90	−27.28 ± 1.07	0.52 ± 0.02	4.84
62.5	56.67 ± 0.76	−20.55 ± 0.58	0.67 ± 0.03	5.62
31.25	49.13 ± 0.5	−26.50 ± 0.48	0.65 ± 0.03	6.01

* *Mean diameter of droplets are expressed in mean ± SE (n = 3)*.

**Figure 5:**
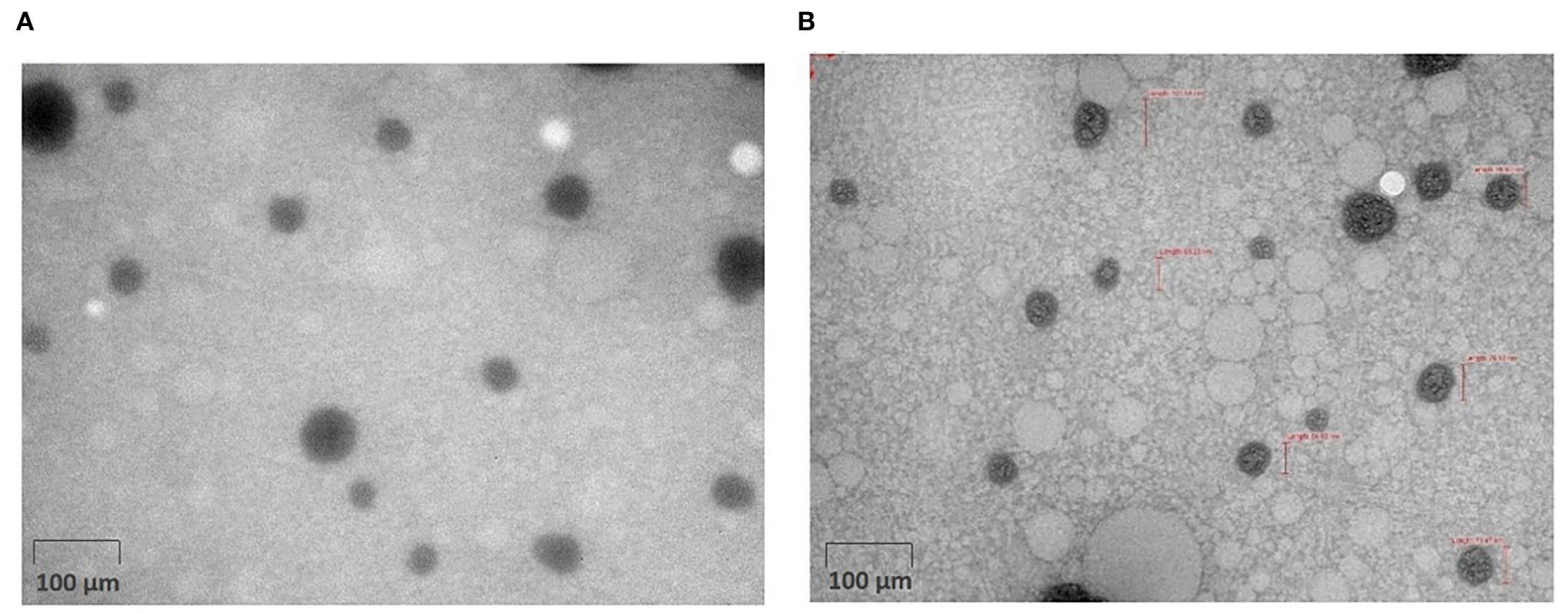
TEM image of 500 μg mL^−1^ concentration of **(A)** NE of EO **(B)** NE of HE.

Morphology and size of the droplets of NEs were visualized under TEM. Figure 5 displayed TEM images of the droplets of NEs of EO and HE. It was quite evident from the TEM images displaying spherical-shaped nanodroplets. The average diameter of the NE of EO droplets was 15.32 nm, which is relatively three times smaller than the average diameter determined obtained from particle size analyzer. Similarly, the average diameter of the NE of lipid-soluble HE droplets was 29.41 nm. However, droplet diameter varied within the range of 12.78 to 38.97 nm. The variation in droplet size could be attributed to the fact that TEM analyses of the droplets in the dry state gave accurate size based on the real morphology of the droplets, whereas average hydrodynamic diameter of the droplets was obtained from the particle size analyzer which was the average size of hydrated micelles. Furthermore, NEs were found to be stable with no phase separation even after 14 days of storage under accelerated storage condition at 54 ± 1°C and at room temperature. The average droplet diameters at 500 μg mL^−1^ concentrations of NEs of EO and HE after 14 days of accelerated storage were 91.22 ± 1.29 nm and 99.41 ± 0.72 nm, respectively. Furthermore, no aggregation of droplets was observed upon TEM analysis, indicating kinetic stability of the emulsions.

### Acaricidal Action on *T. urticae*

Acaricidal activity of CEs and NEs of EO and HE of *P. cablin* against adults of *T. urticae* revealed significant mortality after 24 and 48 h of the treatment (Table 3). Both the CEs were effective after 48 h with the LC_50_ values <170 μg mL^−1^. Of *T. urticae* were found dead along the midrib of the treated leaves. CE-EO was comparatively more toxic than CE-HE. The magnitude of acaricidal efficacy was more after 48 h for the emulsions. Contact toxicity by leaf dip method with respect to time of application might contribute immensely to the actual acaricidal action. As hypotheses, NE-EO and NE-HE were very potent with higher effectiveness, LC_50_ values <100 μg mL^−1^. NE-EO exhibited highest efficacy with the LC_50_ 89.7 μg mL^−1^ and 43.2 μg mL^−1^ after 24- and 48-h exposure, respectively. Likewise, NE-HE was relatively high toxic with their corresponding LC_50_ values of 97.2 μg mL^−1^ and 58.4 μg mL^−1^ after 24- and 48-h exposure.

**Table 3:** Contact toxicity of CEs and NEs against the adults of *T. urticae* after 24 and 48 h of treatment.

**Emulsions**	**Exposure time (h)**	**LC_50_** **(μg mL^−1^)^a^**	**95% Confidence limit μg mL^−1^)**		**Slope ±SE^b^**	**Intercept±SE^c^**	**(χ^2^)^d^**	**df**
			**Lower**	**Upper**				
CE-EO	24	223.6	188.5	293.1	0.85 ± 0.32	−3.39 ± 1.35	29.0	25
	48	134.9	105.3	154.3	1.21 ± 0.13	−1.56 ± 0.97	57.2	34
CE-HE	24	235.6	194.2	301.1	1.03 ± 0.77	−1.86 ± 1.11	25.3	18
	48	168.7	113.5	224.0	0.56 ± 0.09	−3.23 ± 1.32	18.4	12
NE-EO	24	89.7	61.2	113.2	0.95 ± 0.38	−2.60 ± 0.52	32.2	27
	48	43.2	29.6	75.1	0.87 ± 0.25	−4.17 ± 1.92	13.0	9
NE-HE	24	97.2	63.6	144.5	0.44 ± 0.17	−3.27 ± 1.18	39.1	28
	48	58.4	24.7	72.9	1.35 ± 0.70	−1.03 ± 0.69	18.6	22

a *LC_50_ Concentration (μg mL^−1^) at which 50% mortality observed*.

b *Slope at the response of regression equation ± standard error*.

c *Intercept of the regression equation ± SE*.

d *χ^2^, Chi-squared values at different df and probability level (0.05)*.

Fumigant toxicity of the emulsions showed promising action at the sublethal concentrations after 24-h exposure (Table 4). Adults were found dead outside the border of the treated leaves. Among the tested emulsions, NE-EO (LC_50_ 9.3 μg mL^−1^) displayed maximum fumigant action, while similar trend of fumigant action was observed for NE-HE (LC_50_ 13.6 μg mL^−1^). NEs of the oil and non-polar fractions were equally effective in fumigant toxicity, even more potent than the contact toxicity assay.

**Table 4:** Fumigant activity of CEs and NEs against the adults of *T. urticae* after 24 h of treatment.

**Emulsions**	**LC_50_ (μg mL^−1^)^a^**	**95% Confidence limit μg mL^−1^)**		**Slope ±SE^b^**	**Intercept ±SE^c^**	**(χ^2^)^d^**	**df**
		**Lower**	**Upper**				
CE-EO	35.8	21.7	47.9	2.28 ± 0.29	−2.18 ± 0.69	34.6	29
CE-HE	52.4	27.8	72.1	3.94 ± 0.35	−3.25 ± 0.68	22.2	16
NE-EO	13.7	11.6	23.5	4.12 ± 0.35	−2.28 ± 0.65	56.1	30
NE-HE	19.4	12.9	31.6	2.71 ± 0.34	−1.44 ± 0.72	28.3	23

a *LC_50_ Concentration (μg mL^−1^) at which 50% mortality observed*.

b *Slope at the response of regression equation ± standard error*.

c *Intercept of the regression equation ± SE*.

d *χ^2^, Chi-squared values at different df and probability level (0.05)*.

### Insecticidal Action Against *S. litura*

Larvicidal assay of NEs of EO and HE against third instar larvae revealed moderate action. CEs were found less effective with the LC_50_ values >400 μg mL^−1^. However, NEs-EO were effective, performing LC_50_ values 125.8 and 145.9 μg mL^−1^ after 48 and 24 h, respectively (Table 5). Similar findings were noticed for the NEs-HE, which exhibited LC_50_ values of 167.5 μg mL^−1^ and 190.6 μg mL^−1^ after 48- and 24-h exposure, respectively.

**Table 5:** Contact toxicity of CEs and NEs against third instar larvae of *S. litura* using leaf dip assay after 24 and 48 h.

**Emulsions**	**Exposure time (h)**	**LC_50_ (μgmL^−1^)^a^**	**95% Confidence limit μg mL^−1^)**		**Slope ±SE^b^**	**Intercept ±SE^c^**	**(χ^2^)^d^**	**df**
			**Lower**	**Upper**				
CE-EO	24	413.2	395.6	449.5	3.33 ± 0.22	−4.29 ± 0.18	23.7	13
	48	420.5	301.7	442.3	1.48 ± 0.13	−2.69 ± 0.29	39.4	21
CE-HE	24	569.7	423.5	599.8	2.61 ± 0.18	−3.14 ± 0.23	18.8	15
	48	509.1	483.1	524.4	2.25 ± 0.23	−1.25 ± 0.27	19.2	10
NE-EO	24	145.9	137.2	159.7	2.18 ± 0.22	−2.73 ± 0.22	36.5	26
	48	125.8	117.2	144.3	1.17 ± 0.19	−2.28 ± 0.25	44.8	16
NE-HE	24	190.6	172.9	205.3	1.42 ± 0.20	−1.64 ± 0.30	52.3	26
	48	167.5	147.0	191.6	2.22 ± 0.16	−3.39 ± 0.27	17.2	13

a *LC_50_ Concentration (μg mL^−1^) at which 50% mortality observed*.

b *Slope at the response of regression equation ± standard error*.

c *Intercept of the regression equation ± SE*.

d *χ^2^, Chi-squared values at different df and probability level (0.05)*.

**Figure 6:**
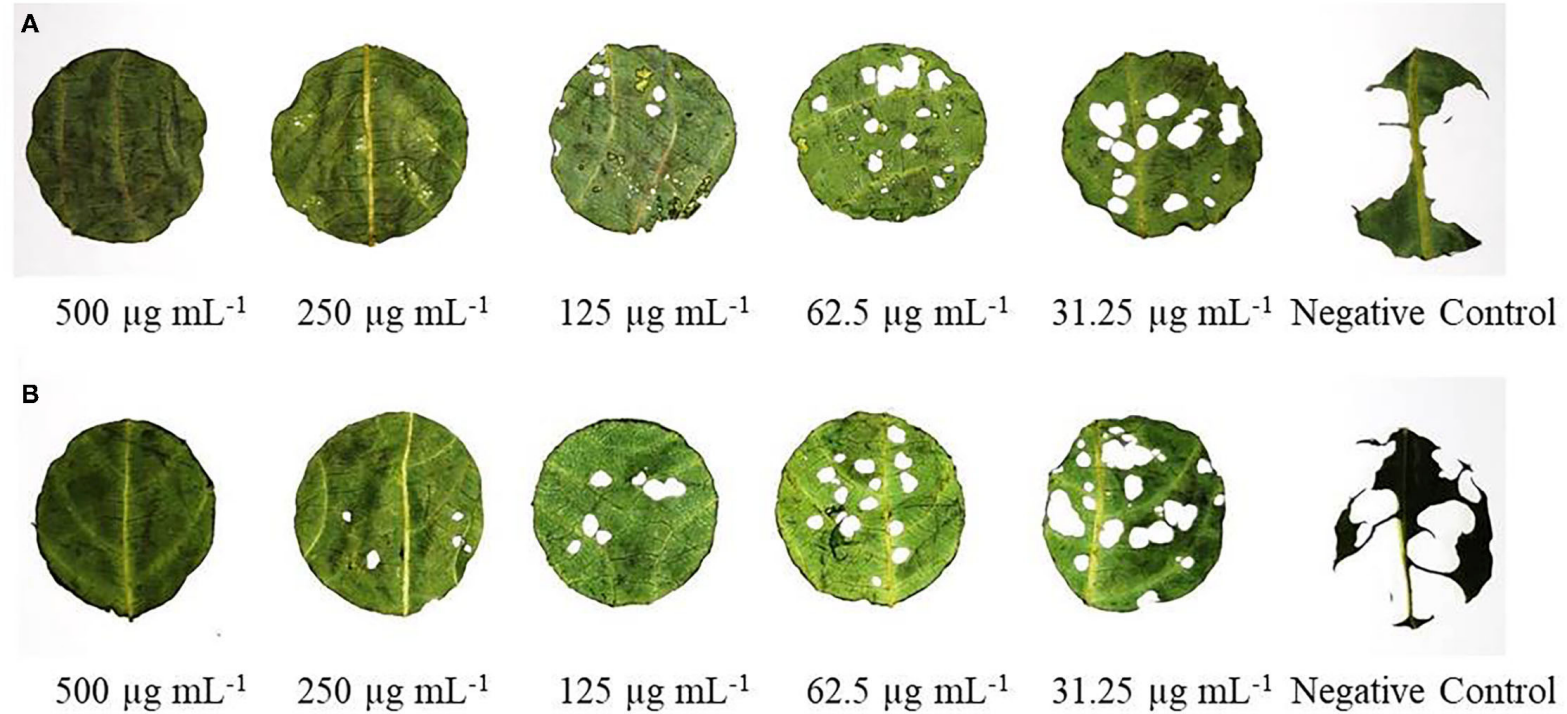
Visual display of antifeedant action of NEs of **(A)** EO and **(B)** HE against *S. litura* third instar larvae after 24 h.

Antifeedant activity of CEs and NEs of EO and HE demonstrated sufficient antifeedant activity at all the test concentrations in both no-choice and choice assays against larvae of *S. litura* (Figure 6). At the highest concentration of 500 μg mL^−1^, CE-EO and CE-HE showed the maximum AI value of 89.75 ± 2.12 and 87.55 ± 2.45, respectively, while NE-EO and NE-HE at the same concentration possessed AI value of 99.21 ± 0.74 and 98.75 ± 1.02, respectively (Table 6). Indeed, antifeedant activity of the CEs has been improved nearly 10% at the higher test concentrations and over 20% at the lower concentrations. On the contrary, NE-EO and NE-HE exhibited FI values of 99.73 ± 1.24 and 97.34 ± 1.0, respectively.

**Table 6:** Evaluation^a^ of CEs and NEs of EO and HE of *P. cablin* for determination of antifeedant index (AI) and feeding index (FI) after 24 h.

**Conc. (μgmL^−1^)**	**CE-EO**		**CE-HE**		**NE-EO**		**NE-HE**	
	**^b^AI (%)**	**^c^FI (%)**	**AI (%)**	**FI (%)**	**AI (%)**	**FI (%)**	**AI (%)**	**FI (%)**
500	89.75 ± 1.12^e^	81.73 ± 1.04^e^	87.55 ± 2.45^e^	77.86 ± 2.38^e^	99.21 ± 0.14^e^	99.73 ± 1.24^d^	98.75 ± 1.02^de^	97.34 ± 1.05^de^
250	85.26 ± 2.45^d^	74.29 ± 3.74^d^	77.75 ± 1.15^d^	63.60 ± 2.45^d^	97.01 ± 1.10^d^	97.86 ± 1.33^d^	98.11 ± 1.68^d^	96.31 ± 2.10^d^
125	70.78 ± 1.39^c^	54.77 ± 2.22^c^	65.43 ± 2.50^c^	48.62 ± 3.57^d^	86.72 ± 2.21^c^	76.56 ± 2.88^c^	84.81 ± 2.30^c^	73.63 ± 3.48^c^
62.5	55.63 ± 1.08^b^	38.53 ± 0.89^b^	52.72 ± 4.22^b^	35.79 ± 2.10^d^	80.08 ± 1.62^b^	66.80 ± 3.06^b^	76.08 ± 2.45^b^	61.39 ± 1.15^b^
31.25	35.66 ± 3.06^a^	21.30 ± 1.33^a^	33.91 ± 3.56^a^	20.42 ± 3.16^d^	72.36 ± 1.74^a^	56.69 ± 1.22^a^	70.29 ± 1.10^a^	54.20 ± 0.74^a^

a *Data are presented as means ± SD*;

b *Antifeedant index (AI) is calculated as AI = (1 – T/C) × 100*,

c *Feeding index (FI) is calculated as FI = (C–T)/(C + T) × 100*.

## Discussion

Comprehensive information on EO of *P. cablin* displayed abundance of either patchoulene or patchouli alcohol (Sundaresan et al., 2012). However, α-guaiene has been reported as the major constituent of *Pogostemon* EO (Tsai et al., 2007). Recently, forty-seven volatile constituents consisting of twenty sesquiterpenes were reported from the EO, which mentioned abundance of curzerene followed by *epi*-cadinol and acetophenone (Kumar et al., 2019b). Exceptionally, aciphyllene and acetophenone are often identified in higher content in the commercially available EO, which was further authenticated to be found as an adulterant, the major constituent of *P. heyneanus* (Murugan et al., 2010). Another literature report, from South Indian sample of *P. cablin*, suggested high content of acetophenone, β-pinene, and *(E)-*nerolidol in the EO (Anjana and Thoppil, 2013). Such variations in chemical compositions of EO could be attributed due to the distillation techniques, associated temperature on extraction, agroclimatic factors along with genetic variations of the planting materials, etc. (Kundu et al., 2013b; Dutta et al., 2020). A recent report suggested quality control and regulation aspects of patchouli EO are highly dependent on the variable composition of patchoulol and other sesquiterpenes (Pandey et al., 2022).

Saponin was used as emulsifier to develop the NEs which certainly contributed in positive manner to enhance the efficacy of the developed emulsions. It is the first report on the use of natural polymer for the preparation of NEs of *Pogostemon* bioactives, though most of the previous studies have been reported on the use of various non-ionic surfactants for the fabrication of NEs of EOs (Balasubramani et al., 2017; Campolo et al., 2017). The wide availability and relatively lower cost of the non-ionic surfactants could be responsible for its higher use. Contrastingly, xanthan gum along with subcritical water has been used to develop oil-in-water NEs (Ahmadi and Jafarizadeh-Malmiri, 2021a). They also suggested the application of natural gums and/or saponin using subcritical water for the preparation of EO-based NEs (Ahmadi and Jafarizadeh-Malmiri, 2021b). Thus, the use of green biopolymer has an edge over the excessive use of conventional emulsifiers in sustainable agriculture. In addition, saponin-like biopolymeric emulsifiers may enhance the biofunctional properties of the component.

In the present study, stable NEs were developed with saponin employing ultrasonication. Low energy subcritical water-based green method has been reported recently for the preparation of thyme oil-in-water NEs (Ahmadi and Jafarizadeh-Malmiri, 2020). The developed NEs exhibited better stability over a period of 14 days. Zeta potential of any emulsion has been recommended as the key indicator for its stability, which is related to electrostatic repulsion among the nanodroplets (Zainol et al., 2012). The stability of the NEs will be maintained with the limited or no coagulation of droplets, and therefore, the colloidal system should retain its droplet diameter in nano size range (<100 nm), resulting in better Brownian motion of the particles in the system (Heydari et al., 2020).

Previously, a homogenization method consisting of multiple adjuvants has been reported for the preparation of NEs of *Pogostemon* EO with non-ionic surfactant, propylene glycol as co-surfactant, and lecithin as emulsifier at 5% concentration; however, concomitant data on the physico-chemical properties have not been generated (Adhavan et al., 2017). Likewise, another study also reported the use of Tween 80 and/or Triton X-100 to stabilize geranium EO maintaining the oil surfactant ratio ranging from 5:1 to 1:5, suggesting better stability of the emulsion with higher amount of surfactant (Jesser et al., 2020). In the present context, NEs were prepared using ultrasound-assisted nano-emulsification of EO and lipid-soluble HE of *P. cablin* leaves with only biosurfactant, saponin at 0.5% concentration, without any use of co-surfactant, and/or co-emulsifier. In addition, better droplet size and emulsion stability of these NEs have been ascertained with rigorous physico-chemical characterizations and process validations.

A preliminary investigation on NEs of *P. cablin* oil (18%) with polyoxyethylene suggested excellent formicidal action on *Atta opaciceps* and *Atta sexdens* (Rocha et al., 2018). Furthermore, *P. cablin* oil-based tincture, candle, and crystal cake have been found effective as mosquito repellent (Das et al., 2016). Recently, patchouli oil has been nano-encapsulated on chitosan and evaluated for enhanced shelf life of maize seeds (Roshan et al., 2022). Recent past, the EO has been reported very effective in repellent action and fumigant toxicity against *T. cinnabarinus* (Cheng et al., 2020). Furthermore, better performance of NEs has been reported in literature against various insects (Badawy et al., 2018; Abdelgaleil et al., 2019). Current study revealed tremendous performance of the NEs over CEs against *T. urticae* both in fumigant and contact toxicity assays. However, required concentration of the emulsions was found to be very less to cause lethal action in fumigant assay, causing suffocation to death due to volatile nature of the chemical constituents. Nevertheless, acaricidal action in contact toxicity has been found satisfactory.

Plant volatiles have been found effective against many agriculturally important pests (Kundu et al., 2013a; Ahluwalia et al., 2014; Keerthiraj et al., 2021). Comprehensive studies have been reported on insecticidal activity of *P. cablin* oil. In the present study, NEs were found to be highly effective to cause larval mortality. Comparative assessment of the insecticidal activity of CEs and NEs revealed higher effectiveness of NEs due to nano sizing of the delivery system with higher surface area and better penetration through insect cell wall. Strong antifeedant and larvicidal action of the plant have been reported in literature against noctuid insects (Huang et al., 2014). NE-EO and NE-HE displayed considerable action with respect to AI and FI against *S. litura* larvae even at the lowest concentration of 31.25 μg mL^−1^. The salient finding of the antifeedant action certainly corroborates the literature report describing more than 80% feeding deterrent action of the oil (Huang et al., 2014). Major chemical components such as patchouli alcohol, α-bulnesene, and α*-g*uaiene in both EO and HE could be responsible for higher efficacy. Though it is unclear whether the most abundant constituent, patchouli alcohol, is only responsible for the acaricidal and insecticidal action, further mechanism of action needs to be studied.

## Conclusions

In summary, patchouli alcohol has been identified as the major constituent of EO and HE of *P. cablin* leaves. Stable NEs have been prepared with only 0.5% saponin either for EO or HE. The NEs of EO and HE were stable even after 30 days at room temperature with satisfactory qualitative characteristics. The results of acaricidal and antifeedant activities have demonstrated promising effect of NEs of EO and HE against *T. urticae* and *S. litura* following a dose-dependent trend, though NEs of EO have been performed slightly better than HE. As far as our literature survey could ascertain, this is the first report on potential activity of NEs of *P. cablin* against *T. urticae*. The output of the study has been well justified with the generation of key information for the development and utilization of NEs. However, future research regarding basic and fundamental studies on the mechanism of action of the compositional terpenoids is still needed for the development of next-generation bio-acaricides.

## Data Availability Statement

The raw data supporting the conclusions of this article will be made available by the authors, without undue reservation.

## Author Contributions

KM conducted the experiments. AK conceptualized the research work, analyzed the data, and wrote the manuscript. AD performed the analysis. SS supervised the experiments. BN analyzed the data and edited the manuscript. All authors contributed to the article and approved the submitted version.

## Funding

This work was supported by the Indian Council of Agricultural Research (ICAR) and the Ministry of Agriculture and Farmers Welfare, Government of India (Grant No. CRSCIARISIL 2014035267), India.

## Conflict of Interest

The authors declare that the research was conducted in the absence of any commercial or financial relationships that could be construed as a potential conflict of interest.

## Publisher's Note

All claims expressed in this article are solely those of the authors and do not necessarily represent those of their affiliated organizations, or those of the publisher, the editors and the reviewers. Any product that may be evaluated in this article, or claim that may be made by its manufacturer, is not guaranteed or endorsed by the publisher.
